# Synchronous mucinous metaplasia and neoplasia of the ovarium and fallopian tube with *STK11* and *KRAS* mutations: a case report

**DOI:** 10.1007/s00428-025-04236-w

**Published:** 2025-09-03

**Authors:** Miroslava Flídrová, Eva Krkavcová, Nikola Hájková, Kristýna Němejcová, Pavel Dundr, Michaela Kendall Bártů

**Affiliations:** Department of Pathology, First Faculty of Medicine, Charles University and General University Hospital, Studničkova 2, 12800 Prague, Czech Republic

**Keywords:** Mucinous metaplasia and neoplasia, Female genital tract, SMMN-FGT, *STK11*, *KRAS*

## Abstract

Synchronous mucinous metaplasia and neoplasia of the female genital tract (SMMN-FGT) is a rare disorder defined as mucinous lesions affecting at least two sites in the female genital tract. We report a case of SMMN-FGT in a Caucasian 65-year-old patient with a right adnexal mass. The patient underwent radical surgery and histological examination showed mucinous ovarian carcinoma combined with mucinous metaplasia of the fallopian tube. The carcinomatous infiltration also affected the left ovary, peritoneum, and omentum. Molecular analysis revealed a shared *STK11* mutation in both the ovarian carcinoma and tubal metaplasia, and other mutations (including *KRAS*) that differed between these tissues. The patient received adjuvant chemotherapy combined with bevacizumab. As there is limited experience with SMMN-FGT, standard diagnostic and treatment protocols have not yet been established. Although association with Peutz-Jeghers syndrome (PJS) was described, our patient had no clinical signs of PJS and the detected *STK11* mutation was likely somatic.

## Introduction

Synchronous mucinous metaplasia and neoplasia of the female genital tract (SMMN-FGT) is a rare mucinous lesion, often with gastric differentiation, first described by Mikami in 2009. SMMN-FGT affects two or more sites in the female genital tract and encompasses a wide spectrum of changes including non-neoplastic metaplasia without nuclear and architectural atypia, benign or borderline neoplasms, and malignant tumours [1–3]. The histogenesis of the process is currently unknown [1]. *STK11* and *KRAS* gene mutations were described in a subset of SMMN-FGT, and some authors reported rare cases of SMMN-FGT occurring simultaneously with Peutz-Jeghers syndrome (PJS), which is also characterised by *STK11* alterations [2, 4–6]. We report a case of SMMN-FGT characterised by a shared *STK11* mutation and distinct *KRAS* gene mutations.

## Material and methods

### Immunohistochemical analysis

Immunohistochemical (IHC) analysis was performed using 4-µm-thick sections of formalin-fixed and paraffin-embedded (FFPE) tissue. The list of antibodies used, their clones, manufacturers, dilution, and staining instruments are summarised in Table 1.

**Table 1:** List of antibodies used for immunohistochemical analysis

Antibody	Clone	Dilution	Producer	Platform	Detection	Results
CDX2	EPR2764Y	1:200	Zytomed Systems GmbH, Berlin, Germany	Ventana BenchMark ULTRA (Roche, Basel, Switzerland)	OptiView	Expression in approximately 20% cells of ovarian carcinoma
CK7	OV-TL 12/30	1:100	Dako, Glostrup, Denmark	Dako Omnis, (Agilent, Santa Clara, CA, USA)	EnVision FLEX (Dako)	Diffuse and strong expression both in ovarian carcinoma and tubal mucinous metaplasia
CK17	E3	1:200	Zeta Corporation, Arcadia, CA, USA	Ventana BenchMark ULTRA (Roche, Basel, Switzerland)	UltraView	Expression in rare cells of ovarian carcinoma
CK20	Ks 20.8	1:50	Dako, Glostrup, Denmark	Dako Omnis, (Agilent, Santa Clara, CA, USA)	EnVision FLEX (Dako)	Expression in rare cells of ovarian carcinoma, negative in tubal mucinous metaplasia
DPC4	B-8	1:200	Zeta Corporation, Arcadia, CA, USA	Dako Omnis, (Agilent, Santa Clara, CA, USA)	EnVision FLEX (Dako) + Linker	Nearly diffuse expression in ovarian carcinoma
ER	SP1	1:200	Zytomed Systems GmbH, Berlin, Germany	Ventana BenchMark ULTRA (Roche, Basel, Switzerland)	OptiView	Negative in ovarian carcinoma, focal weak expression in tubal mucinous metaplasia
PAX8	MRQ-50	RTU	Cell Marque, Rocklin, CA, USA	Ventana BenchMark ULTRA (Roche, Basel, Switzerland)	OptiView	Negative
PR	clone 16	1:100	Novocastra, Leica Biosystems, Wetzlar, Germany	Ventana BenchMark ULTRA (Roche, Basel, Switzerland)	OptiView	Negative
SATB2	EP 281	1:400	Cell Marque, Rocklin, CA, USA	Dako Omnis, (Agilent, Santa Clara, CA, USA)	EnVision FLEX (Dako)	Negative
TTF1	SPT 24	1:200	Biocare Medical, Pacheco, CA, USA	Ventana BenchMark ULTRA (Roche, Basel, Switzerland)	OptiView	Negative
WT1	6F-H2	1:200	BioSB, Santa Barbara, CA, USA	Dako Omnis, (Agilent, Santa Clara, CA, USA	EnVision FLEX	Negative

*ER*, estrogen receptor; *PR*, progesteron receptor

### Molecular analysis

Genomic DNA was isolated from FFPE tissue of the ovarian carcinoma (estimated tumour purity was 70% of tumour cells), from tubal metaplasia (60% of tumour cells), and from a paraaortic lymph node affected by an incidentally detected low-grade marginal zone lymphoma (MZL), using the Quick-DNA/RNA FFPE Miniprep Kit (Zymo Research).

Sequence capture NGS analysis of the DNA was performed using the KAPA HyperPlus kit according to KAPA HyperCap Workflow v3.0 (Roche) and a panel of hybridisation probes against multiple targets of cancer-relevant genes (788 genes or gene parts; 1992 kbp of coding regions; Roche). Paired-end sequencing was carried out on a NextSeq 2000 (Illumina) using P2 reagents. The biostatistical evaluation was performed using CLC Genomics Workbench software (CLC GW; Qiagen, Venlo, The Netherlands) and variants with mutation allele frequency (MAF) of at least 5% were called. Interpretation of the variants was performed as described in a previous study [7].

## Results

### Patient clinical characteristics

A secundiparous Caucasian 65-year-old woman with a personal history of arterial hypertension and thyreopathy was referred to our hospital for a right adnexal mass. CT scan and ultrasound showed a non-homogenous, solid-cystic expansion affecting the right adnexa and measuring up to 48 mm in diameter. There were multiple peritoneal tumour nodules and infiltration of the omentum. Paraaortic lymph nodes were slightly enlarged. Hysterectomy with bilateral adnexectomy, posterior pelvic exenteration, appendectomy, peritonectomy, and omentectomy were performed; however, the surgery was not radical and minimal residual tumour remained on the mesentery.

### Morphological and immunohistochemical findings

Macroscopically, the right ovary was enlarged, 45 × 40 × 30 mm in size, with solid and multicystic structures on section and with papillary tumourous proliferations on the surface. The right fallopian tube was macroscopically normal.

**Fig. 1:**
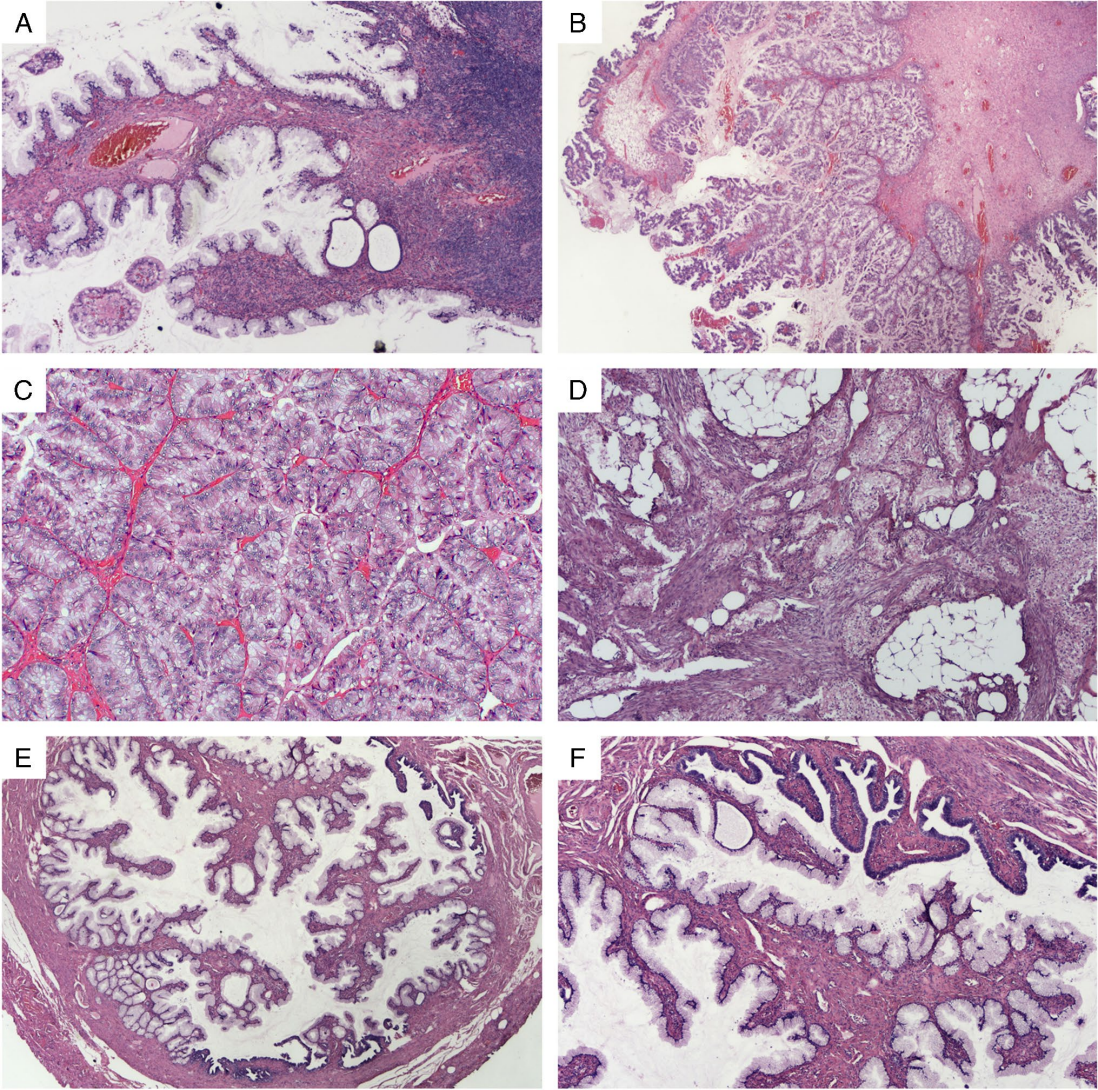
Representative images of the morphological features of the ovarian carcinoma and tubal mucinous metaplasia. All images are stained with haematoxylin–eosin. **A** Part of the ovarian tumour resembling mucinous cystadenoma, × 40 magnification. **B** Ovarian mucinous carcinoma with expansile invasion, × 20 magnification. **C** Higher magnification showing the cytological details of the ovarian mucinous carcinoma, × 100 magnification. **D** Carcinomatous infiltration of the omentum, × 40 magnification. **E** Extensive tubal mucinous metaplasia with the normal serous epithelium being replaced by well-differentiated mucinous epithelium, × 20 magnification. **F** Tubal mucinous metaplasia in detail, × 40 magnification

**Fig. 2:**
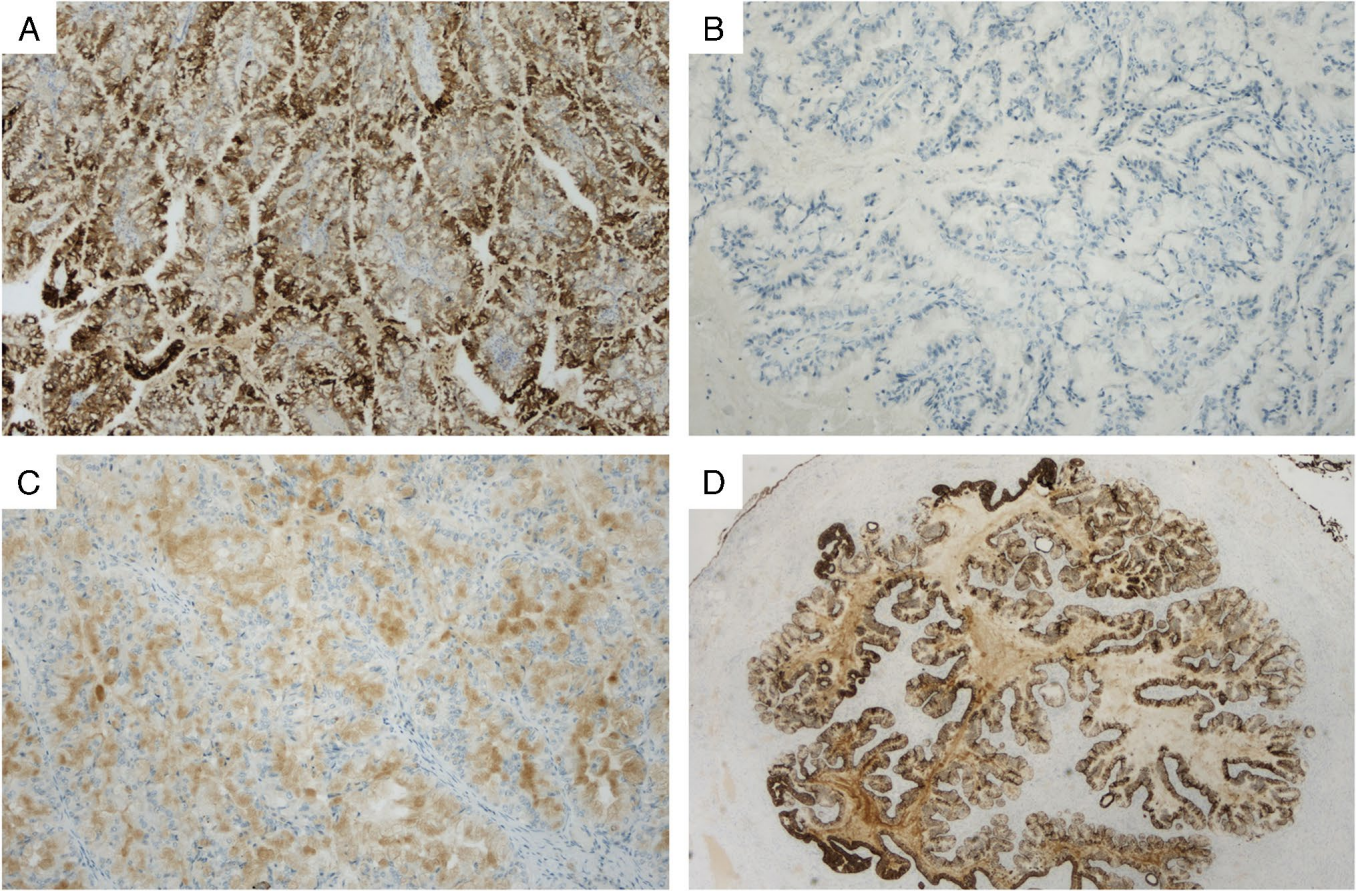
Representative images of immunohistochemical staining. **A** CK7 showing diffuse positivity in the ovarian carcinoma, × 40 magnification. **B** Complete lack of ER expression in the ovarian carcinoma, × 100 magnification. **C** PAX8 showing only nonspecific, weak cytoplasmic staining without nuclear positivity, × 100 magnification. **D** Diffuse positivity of CK7 in the tubal mucinous metaplasia, × 20 magnification

Microscopically, the right ovary contained a mucinous tumour with adenofibromatous arrangement. The tumour was of a heterogeneous appearance, with some areas of benign mucinous cystadenoma, which consisted of bland columnar cells with pale mucinous cytoplasm, without nuclear atypia. These benign areas were intermingled with overtly malignant structures of mucinous carcinoma with complex glandular architecture and with areas of both expansile and infiltrative invasion exceeding 5 mm in size. Despite its normal macroscopic appearance, the right fallopian tube showed extensive mucinous metaplasia with the normal serous epithelium being replaced by well-differentiated mucinous epithelium reminiscent of benign mucinous cystadenoma. The representative images of the morphology are provided in Fig. 1. The results of the immunohistochemical examinations are provided in Table 1, with representative examples of selected immunostains shown in Fig. 2.

The left ovary, omentum, and peritoneum (including rectal and appendiceal serosa) also showed extensive carcinomatous infiltration with mucinous carcinoma. There were no lymph node metastases, but as an additional finding, low-grade MZL was detected in the paraaortic lymph nodes.

### Molecular findings

Both ovarian carcinoma and tubal mucinous metaplasia harboured an identical *STK11* mutation (p.Y60fs), MAF in ovarian carcinoma 26% (read depth × 1137), MAF in tubal metaplasia 27% (× 230). Although both lesions carried *KRAS* mutations, the specific variants differed: G12D (MAF 26%, × 967) was detected in the ovarian carcinoma, whereas G13C (MAF 7%, × 237) and A146V (MAF 14%, × 254) mutations were present in the tubal mucinous metaplasia. Additionally, the ovarian mucinous carcinoma showed pathogenic mutations in *ARID1A* (MAF 17%, × 484), *CFTR* (MAF 41%, × 1448), and *FOXA2* (MAF 7%, × 562), as well as four other variants of uncertain significance (*SF3B1*, *FAT4*, *IL7R*, and *RTEL1*). The list of these variants is available upon request. Both lesions were microsatellite stable. Since we also performed mutation analysis on the paraaortic lymph node’s MZL and did not detect the above-mentioned mutations in this tissue, we consider these mutations to be somatic.

### Clinical follow-up and treatment

Subsequently, neither endoscopic nor imaging methods showed any other possible primary origin of the tumour, therefore the case was ultimately (despite the nonspecific immunoprofile) concluded as primary ovarian mucinous carcinoma with peritoneal spread and with a synchronous mucinous metaplasia of the tubal epithelium. Gastroscopy did not show any abnormality in the upper digestive tract, and colonoscopy revealed only several diminutive polyps on the colonic mucosa. There were no mucocutaneous pigmentations or any other symptoms of PJS. The patient was sent for genetic counselling, which was performed outside our hospital, and the results are not available. After the surgery, the patient received six cycles of adjuvant chemotherapy (paclitaxel-carboplatin) combined with bevacizumab. No further follow-up is available.

## Discussion

SMMN-FGT is a very rare condition, with approximately 35 cases reported in the literature, defined as mucinous lesions affecting at least two anatomical sites of the female reproductive tract, including the cervix, uterus, fallopian tubes, and ovaries [3, 4]. According to the recent data, the mean age at the time of diagnosis is 51 years, and the clinical symptoms are not entirely specific (e.g. abnormal vaginal bleeding, vaginal discharge, abdominal discomfort, or abnormal cervical cytology) [3]. We described a case of SMMN-FGT in a Caucasian 65-year-old woman with a right adnexal mass on imaging, diagnosed with primary ovarian mucinous carcinoma with peritoneal spread and extensive tubal mucinous metaplasia. With the expansion of molecular testing in recent years, specific genetic alterations in SMMN-FGT have been described; however, the data are still limited due to the low number of analysed cases. To our knowledge, fewer than 20 cases were molecularly tested, and they were associated with *STK11* and *KRAS* gene mutations (without further specification of the precise type of the *KRAS* mutation) [2, 4, 6, 8, 9].

These findings are supported by our molecular genetic analysis, which revealed a shared truncating somatic mutation in the *STK11* gene in both the ovarian carcinoma and mucinous tubal metaplasia. Interestingly, the lesions harboured distinct *KRAS* mutations: G12D in the ovary and G13C and A146V in the tubal metaplasia. This fact could reflect early clonal divergence or independent secondary events. According to the available data, it is not yet clear whether SMMN-FGT is a multifocal independent lesion or a spread of a single lesion [3]. To our knowledge, the molecular data on the benign lesions of mucinous metaplasia in SMMN-FGT are very limited, with only a few cases having been tested, in which there were no alterations reported [8, 9]. Ours is the first study to describe the mutational status of both the tumour and the metaplastic lesion. Additional pathogenic mutations (*ARID1A*, *CFTR*, and *FOXA2*) and variants of uncertain significance (*SF3B1*, *FAT4*, *IL7R*, and *RTEL1*) were found only in the ovarian tumour, supporting further clonal evolution in the malignant component. However, their clinical significance remains uncertain. Some authors also described extremely rare cases of SMMN-FGT occurring in patients with Peutz-Jeghers syndrome [4–6, 8, 10–12].

The main differential diagnosis of SMMN-FGT is a metastatic spread of mucinous adenocarcinomas from other primary origins (especially from the gastrointestinal tract), which can show very similar morphological features [3]. Because SMMN-FGT often exhibits gastric-type differentiation, the immunoprofile may not be completely specific and the distinction of these entities is sometimes difficult, therefore correlation with clinical and imaging findings is necessary.

Given the rarity of SMMN-FGT, standard diagnostic/treatment protocols or prognostic factors have not yet been established, although recent data suggest that the presence of *STK11* alterations is associated with significantly worse prognosis [2, 4, 8, 9, 13, 14]. The prognosis also seems to depend on the stage of the co-existing adenocarcinoma, and the benign metaplasia/borderline changes do not affect the patient outcome [1]. In our case, adjuvant chemotherapy in combination with bevacizumab was administered, but postoperative clinical follow-up was not available.

## Conclusion

We described a case of SMMN-FGT, which is a very rare entity with limited experience. In all multifocal mucinous lesions of the female genital tract, the possibility of SMMN-FGT should be considered, and the diagnosis may be supported by molecular testing. Due to the rarity of the condition, standard diagnostic and treatment protocols, as well as prognostic factors, have not yet been defined, and further research is necessary. Association with PJS should be ruled out in all patients.

## Data Availability

All data generated or analysed during this study are included in this published article. Raw sequencing data are available upon request.

## References

[1] Mikami Y, Kiyokawa T, Sasajima Y et al (2009) Reappraisal of synchronous and multifocal mucinous lesions of the female genital tract: a close association with gastric metaplasia. Histopathology 54(2):184–191. 10.1111/j.1365-2559.2008.03202.x 19207943 10.1111/j.1365-2559.2008.03202.x

[2] Wang R, Yu H, Liu M, Hao T, Wang X, Cao L (2023) Synchronous mucinous metaplasia and neoplasia of the female genital tract with both pulmonary metastases and STK11/KRAS gene mutations: a case report. Front Oncol 13:1246821. 10.3389/fonc.2023.1246821 38023125 10.3389/fonc.2023.1246821 PMC10679390

[3] Xu H, Chen Y, Zhou S et al (2022) Synchronous mucinous metaplasia and neoplasia of the female genital tract (SMMN FGT): a case report and literature review. Exp Ther Med 25(2):73. 10.3892/etm.2022.11772 36684649 10.3892/etm.2022.11772 PMC9843491

[4] Zhou Y, Wang X, Li Y, et al. When synchronous mucinous metaplasia and neoplasia of the female genital tract and peutz-jeghers syndrome meet: a case report and literature reviews. *BMC Womens Health*. Jun 28 2024;24(1):375. 10.1186/s12905-024-03184-y 10.1186/s12905-024-03184-y PMC11212150 38937781

[5] Mangili G, Taccagni G, Garavaglia E, Carnelli M, Montoli S (2004) An unusual admixture of neoplastic and metaplastic lesions of the female genital tract in the Peutz-Jeghers syndrome. Gynecol Oncol 92(1):337–342. 10.1016/j.ygyno.2003.10.005 14751181 10.1016/j.ygyno.2003.10.005

[6] Bennett JA, Young RH, Howitt BE et al (2021) A distinctive adnexal (usually paratubal) neoplasm often associated with Peutz-Jeghers syndrome and characterized by STK11 alterations (STK11 adnexal tumor): a report of 22 cases. Am J Surg Pathol 45(8):1061–1074. 10.1097/PAS.0000000000001677 33534223 10.1097/PAS.0000000000001677 PMC8277663

[7] Dundr P, Bartu M, Bosse T et al (2023) Primary mucinous tumors of the ovary: an interobserver reproducibility and detailed molecular study reveals significant overlap between diagnostic categories. Mod Pathol 36(1):100040. 10.1016/j.modpat.2022.100040 36788074 10.1016/j.modpat.2022.100040

[8] Kuragaki C, Enomoto T, Ueno Y et al (2003) Mutations in the STK11 gene characterize minimal deviation adenocarcinoma of the uterine cervix. Lab Invest 83(1):35–45. 10.1097/01.lab.0000049821.16698.d0 12533684 10.1097/01.lab.0000049821.16698.d0

[9] Nagahama K, Yamanaka S, Nakayama T et al (2013) A case of synchronous mucinous metaplasia and neoplasia of the female genital tract without an STK11 or KRAS mutation. Gynecol Oncol Case Rep 5:4–5. 10.1016/j.gynor.2013.02.005 24371681 10.1016/j.gynor.2013.02.005 PMC3862322

[10] Bronte Anaut M, Arredondo Montero J, Fernandez Seara MP, Guarch Troyas R (2023) Gastric-phenotype mucinous carcinoma of the fallopian tube with secondary ovarian involvement in a woman with Peutz-Jeghers syndrome: a case report. Int J Surg Pathol 31(1):92–97. 10.1177/10668969221095264 35466733 10.1177/10668969221095264

[11] Young RH, Scully RE (1988) Mucinous ovarian tumors associated with mucinous adenocarcinomas of the cervix. A clinicopathological analysis of 16 cases. Int J Gynecol Pathol 7(2):99–111. 10.1097/00004347-198805000-00001 2840404 10.1097/00004347-198805000-00001

[12] Song SH, Lee JK, Saw HS et al (2006) Peutz-jeghers syndrome with multiple genital tract tumors and breast cancer: a case report with a review of literatures. J Korean Med Sci 21(4):752–757. 10.3346/jkms.2006.21.4.752 16891826 10.3346/jkms.2006.21.4.752 PMC2729904

[13] Lu J, Gao L, Yu H, Xia L, Guo X, Zheng F (2019) A patient from Zhejiang, China, with synchronous mucinous metaplasia and neoplasms of the female genital tract: a case report. J Obstet Gynaecol Res 45(7):1382–1385. 10.1111/jog.13959 30993814 10.1111/jog.13959

[14] Hirose S, Murakami N, Takahashi K et al (2020) Genomic alterations in STK11 can predict clinical outcomes in cervical cancer patients. Gynecol Oncol 156(1):203–210. 10.1016/j.ygyno.2019.10.022 31757465 10.1016/j.ygyno.2019.10.022

